# Post-transcriptional regulation of 2-acetyl-1-pyrroline (2-AP) biosynthesis pathway, silicon, and heavy metal transporters in response to Zn in fragrant rice

**DOI:** 10.3389/fpls.2022.948884

**Published:** 2022-08-17

**Authors:** Muhammad Imran, Sarfraz Shafiq, Sara Ilahi, Alireza Ghahramani, Gegen Bao, Eldessoky S. Dessoky, Emilie Widemann, Shenggang Pan, Zhaowen Mo, Xiangru Tang

**Affiliations:** ^1^Department of Crop Science and Technology, College of Agriculture, South China Agricultural University, Guangzhou, China; ^2^Scientific Observing and Experimental Station of Crop Cultivation in South China, Ministry of Agriculture, Guangzhou, China; ^3^Department of Anatomy and Cell Biology, University of Western Ontario, London, ON, Canada; ^4^Department of Economics, Lahore College for Women University, Lahore, Pakistan; ^5^Department of Plant Genetic Transformation, Agricultural Genetic Engineering Research Institute, Agricultural Research Center, Giza, Egypt; ^6^Institut de Biologie Moléculaire des Plantes, CNRS-Université de Strasbourg, Strasbourg, France

**Keywords:** fragrant rice, 2-acetyl-1-pyrroline, gene expression, zinc, alternative splicing, heavy metals, post-transcription

## Abstract

Fragrant rice (*Oryza sativa* L.) has a high economic and nutritional value, and the application of micronutrients regulates 2-acetyl-1-pyrroline (2-AP) production, which is responsible for aroma in fragrant rice. Alternative splicing (AS) is an important post-transcriptional regulatory mechanism to generate transcript variability and proteome diversity in plants. However, no systematic investigation of AS events in response to micronutrients (Zn) has been performed in fragrant rice. Furthermore, the post-transcriptional regulation of genes involved in 2-AP biosynthesis is also not known. In this study, a comprehensive analysis of AS events under two gradients of Zn treatment in two different fragrant rice cultivars (Meixiangzhan-2 and Xiangyaxiangzhan) was performed based on RNA-seq analysis. A total of 386 and 598 significant AS events were found in Meixiangzhan-2 treated with low and high doses of Zn, respectively. In Xiangyaxiangzhan, a total of 449 and 598 significant AS events were found in low and high doses of Zn, respectively. Go analysis indicated that these genes were highly enriched in physiological processes, metabolism, and cellular processes in both cultivars. However, genotype and dose-dependent AS events were also detected in both cultivars. By comparing differential AS (DAS) events with differentially expressed genes (DEGs), we found a weak overlap among DAS and DEGs in both fragrant rice cultivars indicating that only a few genes are post-transcriptionally regulated in response to Zn treatment. We further report that Zn differentially regulates the expression of 2-AP biosynthesis-related genes in both cultivars and Zn treatment altered the editing frequency of single nucleotide polymorphism (SNPs) in the genes involved in 2-AP biosynthesis. Finally, we showed that epigenetic modifications associated with active gene transcription are generally enriched over 2-AP biosynthesis-related genes. Similar to the 2-AP pathway, we found that heavy metal transporters (genes related to silicon, iron, Zn and other metal transport) are also regulated at transcriptional and post-transcriptional levels in response to Zn in fragrant rice. Taken together, our results provide evidence of the post-transcriptional gene regulation in fragrant rice in response to Zn treatment and highlight that the 2-AP biosynthesis pathway and heavy metal transporters may also be regulated through epigenetic modifications. These findings will serve as a cornerstone for further investigation to understand the molecular mechanisms of 2-AP biosynthesis and regulation of heavy metal transporters in fragrant rice.

## Introduction

Fragrant rice (*Oryza sativa* L.) is eminent around the world due to its unique aroma and the significant growth has been seen in the price and demand (Sakthivel et al., 2009; Hashemi et al., 2013). Nevertheless, the biosynthesis of aromatic compounds is an extremely complex process and until now 200 volatile compounds have been identified in fragrant rice (Champagne, 2008). Recently, 2-acetyl-1-pyrroline (2-AP) has been recognized as a significant distinctive aromatic compound which is responsible for the aroma production in the fragrant rice (Poonlaphdecha et al., 2016; Wakte et al., 2017). The biosynthesis of 2-AP is processed by two pathways, one is enzymatic, and the other is non-enzymatic. During the non-enzymatic pathway, proline, glutamate, and ornithine are converted to 1-pyrroline-5-carboxylate (P5C) *via* the Δ1-pyrroline-5-carboxylate synthetase (P5CS), proline dehydrogenase (PDH), and ornithine aminotransferase (OAT) enzymes and Δ1-pyrroline is further converted to 2-AP *via* a non-enzymatic reaction of methylglyoxal (Kaikavoosi et al., 2015). However, enzymatic pathways are dependent on Betaine Aldehyde Dehydrogenase (BADH), which is responsible for the conversion of gamma-aminobutyl aldehyde (GABald) into GABA (Chen et al., 2008). The *BADH2* gene expression inhibits 2-AP production in non-fragrant rice, whereas *BADH2* gene is inactive in fragrant rice. The non-active *BADH2* gene in fragrant rice converts the GABald to 1-pyrroline, which leads to the biosynthesis of the 2-AP (Chen et al., 2008). Further, the biosynthesis of 2-AP is positively correlated with the proline and γ-aminobutyric acid (GABA) foliar application at the heading stage in fragrant rice (Mo et al., 2016). Surprisingly, it also has been reported that the exogenous application of specific micronutrients to fragrant rice can significantly enhance the 2-AP content of the rice grain. Using fragrant rice as an example, it has been demonstrated that the application of manganese increased the biosynthesis of the 2-AP (Li et al., 2016). Another study found that the use of silicon could boost the chlorophyll and the 2-AP content of the fragrant rice (Mo et al., 2017). Thus, an exogenous application of microelements in fragrant rice could be a highly effective method of upregulating 2-AP biosynthesis.

As a micronutrient, zinc (Zn) is essential for plant growth and also has vital physiological and biochemical functions (da Silva and Santos, 2017). In humans, zinc deficiency can result in physical deterioration, loss of vision, lower immune function and disease resistance (Adamo et al., 2019). As a result, delivering adequate amounts of essential minerals in food has emerged as a critical goal in the fight against “hidden hunger” (Hirschi, 2009). To fulfill this demand, micronutrient foliar treatment provides a quick and reliable solution, and it has been successfully implemented in a variety of crops, such as rice, wheat and beans (White and Broadley, 2011; Li et al., 2018). Interestingly, it has been reported that *in vitro* application of Zn on the detached fragrant rice panicles might increase the 2-AP content due to increased proline content and proline dehydrogenase activity (Mo et al., 2016). Moreover, the role of rice flag leaves in grain filling, biosynthesis, and mineral translocation is already established (Narayanan et al., 2007; Sperotto et al., 2013). These studies indicate that aromatic traits of fragrant rice and Zn biofortification could be achieved together. Nevertheless, the mechanism by which foliar Zn treatment influences 2-AP biosynthesis at the transcriptional level is unknown.

The expression of genes is highly regulated at the transcriptional and post-transcriptional levels, which occurs at the level of pre-messenger RNA (mRNA) processing (capping, splicing and polyadenylation), mRNA stability and mRNA translation (Breitbart et al., 1987; Floris et al., 2009). In addition to the transcriptional control of gene expression, other less studied regulatory mechanisms are essential. Among them, alternative splicing (AS), the differential processing of introns and exons in pre-mRNAs to produce multiple transcript isoforms per gene, is the most important contributor to transcriptome diversification in both plants and animals (Irimia and Blencowe, 2012; Reddy et al., 2013; Staiger and Brown, 2013). Alternative splicing generates multiple functional mRNAs from one single gene which consequently expands transcript variability and proteome diversity in organisms (Breitbart et al., 1987; Staiger and Brown, 2013). There are five most common types of AS; Skipping exon (SE), Alternate 5′ or 3′, Retention intron (RI), and Mutually exclusive exons (MXE) (Breitbart et al., 1987). Skipping exon (SE) is a type of AS in which certain exons are removed from mRNA. Alternate 5′ or 3′ splicing (A5′ SS and A3′ SS) is a type of AS in which AS can be mediated by the joining of exons at alternative 5′ or 3′ splice sites. Retention intron (RI) is a type of AS in which non-coding portions of a gene are retained in the final mRNA. Mutually exclusive exons (MXE) is a type of AS in which one and only one exon from a gene is included in the mRNA. AS contributes to environmental adaptation and phenotypic plasticity in organisms and AS has been reported to play an important role in plants in response to abiotic stresses, such as temperature, nutrient deficiency, and salt stresses (Chang et al., 2014; Ding et al., 2014; Filichkin et al., 2015; He et al., 2015; Dong et al., 2018). Therefore, the precision and efficiency of alternative splicing are critical factors in gene function. Furthermore, the identification of high-impact DNA polymorphisms, such as single-nucleotide polymorphisms (SNPs), insertions/deletions (InDels), and structural variants (SVs) inside coding areas could alter the gene expression patterns (Mortazavi et al., 2022), and the next-generation sequencing (NGS) of transcriptome may facilitate in this regard. Epigenetic modifications, another example of post-transcriptional modifications, are important for crop improvement and other plant functions (Bannister and Kouzarides, 2011; Berr et al., 2011; Shafiq and Khan, 2015). Among the histone modifications, methylations of histone H3 at lysine 4 (H3K4) and lysine 36 (H3K36) are generally associated with transcriptional activation, whereas methylations of histone H3 at lysine 9 (H3K9) and lysine 27 (H3K27) are associated with transcriptional repression (Bannister and Kouzarides, 2011; Berr et al., 2011; Zhao et al., 2015). Furthermore, histone acetylation is associated with the transcriptional activation (Berr et al., 2011), whereas DNA methylation is associated with the transcriptional repression (Zhang et al., 2018). DNA methylation occurs in plants in the CG, CHG, and CHH sequence contexts (where H is A, C, or T), and it is highly enriched over heterochromatic transposable elements (TEs) and repeats, where it plays a significant role in suppressing their transcriptional activity (Transcriptional Gene Silencing (TGS). When DNA methylation occurs in gene regulatory areas, it can potentially cause TGS. Furthermore, it has been demonstrated that methylation of intronic TEs and repeats affects mRNA processing mechanisms such as alternative splicing and alternative polyadenylation (Zhang et al., 2018). However, the post-transcriptional regulatory role of AS in response to Zn treatment is not known. Furthermore, whether 2-AP biosynthesis in fragrant rice is post-transcriptionally regulated is also not known.

High-throughput sequencing strategies have now enabled us to understand the regulatory role of AS in plants. In our previous study, transcriptomic analysis was performed to understand the function of the Zn-induced transcriptional cascades regulating 2-AP biosynthesis in fragrant rice (Bao et al., 2021; Imran et al., 2022). Here, we identified the AS events in two fragrant rice cultivars in response to low and high doses of Zn application. We further compared the expression of genes involved in 2-AP biosynthesis and heavy metal transport in two fragrant rice cultivars and investigated their regulation by AS and SNPs in response to Zn treatment. Our research provides valuable insights into the molecular regulatory mechanisms that regulate 2-AP biosynthesis and heavy metal transport in fragrant rice.

## Materials and methods

### Plant material and growth conditions

The seeds of two fragrant rice varieties, i.e., “Meixiangzhan-2” (M) and “Xiangyaxiangzhan” (X) were obtained from South China Agricultural University, Guangzhou, China. The experiment was conducted in the pots under controlled conditions in the greenhouse (113°21′E, 23°14′N) at the South China Agricultural University. Three Zn concentrations were used in the experiments; without Zn CK: 0 mg L^–1^, T1: 14 mg L^–1^, and T2: 30 mg L^–1^ and denoted as MT1, MT2 for Meixiangzhan-2 and XT1, XT2 for Xiangyaxiangzhan cultivar. These treatment values were determined using data from our previously published research (Mo et al., 2016). At the heading stage (from 9:00 to 11:00 am), each treatment used a 1 L amount of zinc solution in the jet pump to guarantee that each leaf was sprayed. At 15 days after the heading stage (AHS), the flag leaf samples were harvested early morning (from 8:00 to 9:00 am) and snap frozen in the liquid nitrogen. Samples were later stored at −80°C.

### RNA sequencing, differentially expressed genes analysis and filtering of 2-AP biosynthesis pathway genes, silicon and heavy metal transporters

RNA sequencing was performed on 15d AHS of fragrant rice flag leaves and both fragrant rice cultivars had two biological replications. The RNA was extracted, and the cDNA library was produced following the manufacturer’s instructions using an Illumina HiSeqTM RNA Sample Prep Kit (Illumina, United States). The quality of the raw reads produced by sequencing was evaluated using the FastQC version (Andrews, 2010). The mapping of high-quality filtered reads against the reference genome acquired from *Oryza sativa* subsp. indica cv. Minghui 63^1^ was used for mapping reads (Zhang et al., 2016) by using HISAT2 version 2.0.5 (Pertea et al., 2016). StringTie version 1.3.3b to assemble the known genes and transcripts from available following reference genome StringTie version 1.3.3b was used (Pertea et al., 2015). Cufflinks version 2.2.1 was used to determine the gene transcripts and standardized as Fragments Per Kilobase Million (FPKM) (Trapnell et al., 2010; Li and Dewey, 2011).

Gene expression with a false discovery rate (FDR) < 0.05 and log2ratio > 1 (twofold change) in the comparative analysis was recognized as a significantly differentially expressed gene (DEG) in the comparative analysis (Li and Dewey, 2011). Then from the DEGs, we filtered the genes regulating 2-AP biosynthesis pathway, Silicon, Iron, Zinc and others heavy metal transporters in response to Zn treatment.

### Alternative splicing analysis

The results of Tophat (Trapnell et al., 2009) included all alternative splicing information. First the alternative splicing sites with less than 5 reads were filtered out. Then the alternative splicing sites were mapped to known alternative splicing sites (1 bp error was allowable) to identify known alternative splicing sites. Finally, the unmapped new alternative splicing sites were classified.

### Single nucleotide polymorphism analysis

The Genome Analysis Toolkit (GATK) (Van der Auwera et al., 2013) was used for calling variants of transcripts, and ANNOVAR (Wang et al., 2010) was used for SNP/InDel annotation. The function, genome site and type of variation of SNPs were also analyzed. We use the following criteria to screen reliable editing sites from SNP sites; (1) Removing the low-quality SNPs while calling SNP by GATK. (2) Correcting the SNPs around the InDel region. (3) Choosing non-overlapping SNPs in UTR and Exon region. (4) Choosing SNPs with reference reads > 2 and variate reads > 3. (5) Choosing SNPs with a mutation frequency between 0.1 and 0.9.

### Gene ontology analysis of differential alternative splicing genes

The gene names containing DAS sites were converted to MSU format using the Rice Information Gateway website^2^ (Zhang et al., 2016). The RiceNetDB^3^ (Liu et al., 2013) was then used to perform gene ontology (GO) enrichment analysis and the results were plotted with ggplot2 v3.3.5 in R v4.1.0.

### Analysis of epigenetic modifications

The coverage files (bigwig format) of rice epigenetic modifications were downloaded from NCBI GEO under accession number GSE142570 to use as inputs for plotProfile function from Deeptools v3.5.1 to assess the coverages of genes involved in the 2-AP biosynthesis pathways.

### Real-time quantitative RT-PCR

Total RNA was extracted using the Tiangen RNA kit and Hiscript II QRT SuperMix was used for real-time quantitative RT-PCR (qRT-PCR) (Guangzhou, China). The Nanodrop 2000 was used to determine the quality and amount of RNA. To perform the qRT-PCR, SYBR Premix Ex Taq (Takara Bio Inc.) was used. The Option Real-Time PCR System CFX96 apparatus was utilized for the real-time quantitative qRT-PCR (Bio-Rad, CA, United States). We adjusted the qPCR machine at 95°C for 30 s, 95°C for 5 s, 57°C for 30 s, and 40 cycles for PCR reaction. *Actin* was as internal control and Primer5 was used to design the primers. All primers were listed in Supplementary Table 1.

### Statistical analyses

Data analysis was performed on Office 2019 (Microsoft). SPSS 25 was used to analyze variance (Analytical Software, Chicago).

## Results

### Zn differently induces alternative splicing events in fragrant rice cultivars

**FIGURE 1:**
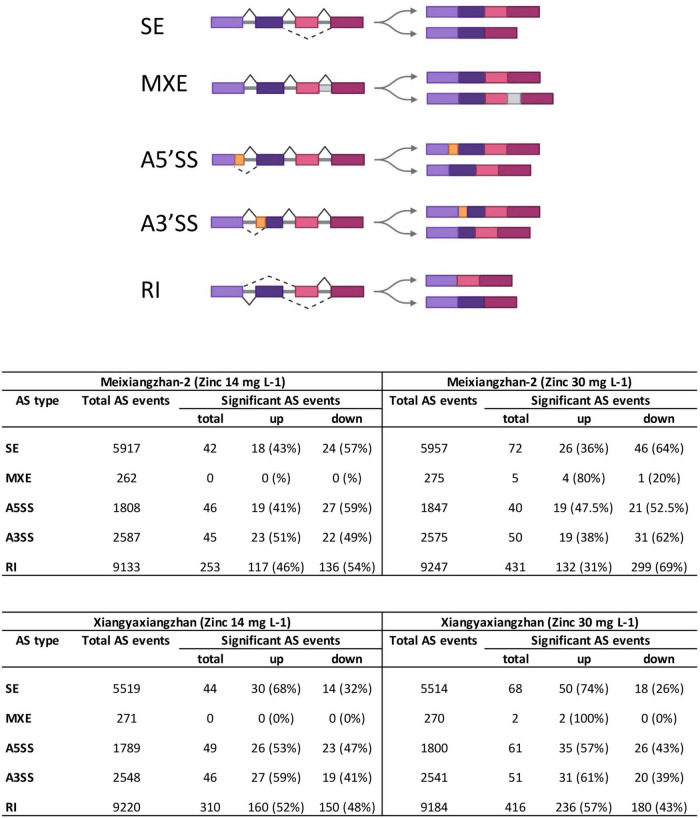
The number of all types of alternative splicing (AS) events in Meixiangzhan-2 and Xiangyaxiangzhan cultivars in response to different levels of Zn treatment. An overview of 5 different types of AS is presented at the top (created with Biorender). SE, skipped exon; MXE, mutually exclusive exons; A5SS, alternative 5′ splice site; A3SS, alternative 3′ splice site; RI, retained intron.

To profile the AS events in response to Zn treatment, RNA-seq data were re-analyzed in two fragrant rice cultivars (Bao et al., 2021). Meixiangzhan-2 treated with 14 mg L^–1^ Zn (MT1) exhibited a total of 19,707 AS events as compared to the control, while Meixiangzhan-2 treated with 30 mg L^–1^ Zn (MT2) exhibited a total of 19,901 AS events as compared to the control (Figure 1 and Supplementary Table 2). We found that Meixiangzhan-2 showed dose-dependent changes in the number of significant AS sites as compared to the control. As compared to the control, a total of 386 and 598 significant AS sites were found in MT1 and MT2, respectively. Among 386 total significant AS sites found in MT1, 177 AS events (45%) were increased as compared to the control, while 209 AS events (54%) were decreased. Among 598 total significant AS sites found in MT2, 200 AS events (33%) were increased as compared to the control, while 398 AS events (67%) were decreased. We further compared AS events between MT1 and MT2 and found a total of 347 differential AS events (Supplementary Table 3). These results indicate that significant AS events decrease with the increase of the dose of Zn in Meixiangzhan-2. Xiangyaxiangzhan treated with 14 mg L^–1^ Zn (XT1) exhibited a total of 19,347 AS events as compared to the control, while Xiangyaxiangzhan treated with 30 mg L^–1^ Zn (XT2) exhibited a total of 19,309 AS events as compared to the control. Similar to Meixiangzhan-2, Xiangyaxiangzhan also showed dose-dependent changes in the number of significant AS sites as compared to the control. As compared to the control, a total of 449 and 598 significant AS sites were found in XT1 and XT2, respectively. Among 449 total significant AS sites found in XT1, 243 AS events (54%) were increased as compared to the control, while 206 AS events (46%) were decreased. Among 598 total significant AS sites found in XT2, 354 AS events (60%) were increased as compared to the control, while 244 AS events (40%) were decreased. We also compared AS events between XT1 and XT2 and found a total of 350 differential AS events (Supplementary Table 3). Contrary to MT1 and MT2, significant AS sites increase with the increase of the dose of Zn in Xiangyaxiangzhan.

We next classified the total and significant AS events into five categories as illustrated in Figure 1. We found that majority of the total and significant AS events belong to RI in both Meixiangzhan-2 and Xiangyaxiangzhan (Figure 1). However, the lowest number of total and significant AS events belong to MXE in both Meixiangzhan-2 and Xiangyaxiangzhan. Interestingly, the number of significant AS events belonging to these five classes was generally decreased in Meixiangzhan-2 but increased in Xiangyaxiangzhan in a dose-dependent manner as compared to their controls. For example, MT1 showed a 46% increase and a 54% decrease in RI events as compared to the control, while MT2 showed a 31% increase and a 69% decrease in RI events. Whereas XT1 showed a 52% increase and a 48% decrease in RI events, and XT2 showed a 57% increase and a 43% decrease in RI events as compared to the control.

### Overlap and gene ontology analysis of differentially alternative splicing sites in both fragrant rice cultivars

**FIGURE 2:**
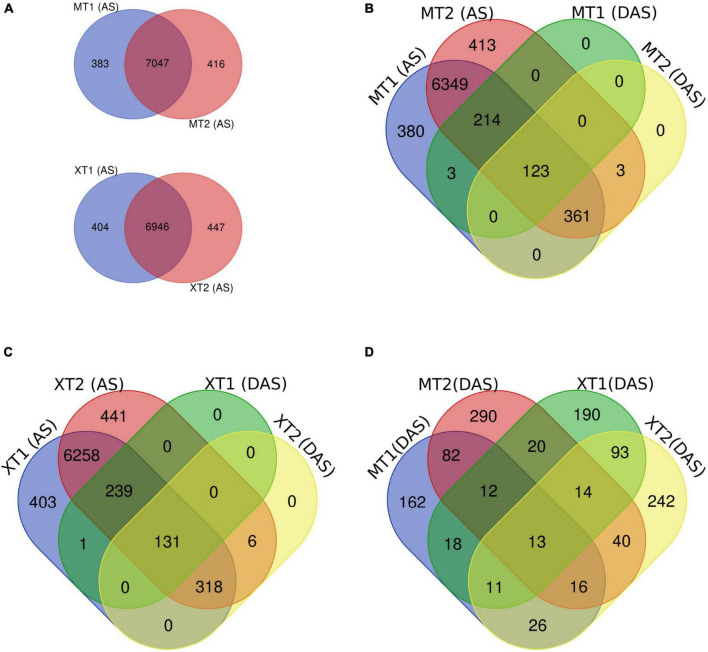
Overlap of differentially alternative spliced (DAS) events with a total of unique AS sites in Meixiangzhan-2 (M) and Xiangyaxiangzhan (X) cultivars in response to different levels of Zn treatment. **(A)** Venn diagram representing the overlap of unique AS sites in response to low (T1) and high dose (T2) of Zn in both cultivars. **(B,C)** Venn diagram representing the overlap between unique AS sites and DAS events in response to different doses of Zn in both cultivars. **(D)** Venn diagram representing the overlap of DAS sites in both cultivars in response to different doses of Zn.

**FIGURE 3:**
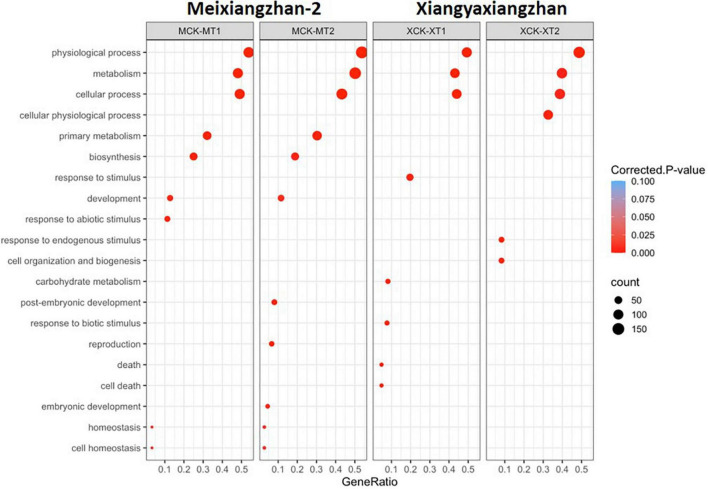
GO classification of differentially alternative spliced (DAS) events in Meixiangzhan-2 (M) and Xiangyaxiangzhan (X) cultivars in response to different levels of Zn treatment. Colors indicate the *P*-values from Fisher’s exact test, and dot size is proportional to the number of genes (the genes that exhibited DAS events) in the given pathway.

First, we overlapped the total AS events in both Meixiangzhan-2 and Xiangyaxiangzhan and found a ∼95% overlap of unique AS events between MT1 and MT2, and XT1 and XT2 (Figure 2A). Next, we overlapped total AS events with DAS events in both Meixiangzhan-2 and Xiangyaxiangzhan cultivars under different levels of Zn treatment. In Meixiangzhan-2, a total of 123 unique DAS sites were found common between MT1 and MT2, whereas 214 DAS sites were found specific to MT1, and 361 DAS sites were found specific to MT2 (Figure 2B). In Xiangyaxiangzhan, a total of 131 unique DAS sites were found common between XT1 and XT2, whereas 239 DAS sites were found specific to XT1, and 381 DAS sites were found specific to XT2 (Figure 2C). These observations indicate that different doses of Zn treatment differently affect the DAS events in both Meixiangzhan-2 and Xiangyaxiangzhan cultivars. Furthermore, we investigated the genotype-dependent Zn induced DAS sites by comparing the Meixiangzhan-2 and Xiangyaxiangzhan cultivars (Figure 2D). We considered a common DAS site if present in both cultivars (irrespective of Zn dose) and found that a total of 161 DAS sites are common between the Meixiangzhan-2 and Xiangyaxiangzhan cultivars. Only 13 DAS sites are common between the two genotypes in a dose-dependent manner of Zn. Consistent with these observations, GO analysis of DAS sites demonstrated that physiological processes, metabolism and cellular process are commonly enriched pathways in both cultivars under different regimes of Zn treatment (Figure 3). However, we also observed genotype-dependent enrichment of pathways. For example, primary metabolism and biosynthesis are enriched in Meixiangzhan-2 but not in Xiangyaxiangzhan. Furthermore, we also observed a dose-dependent enrichment of pathways in each cultivar. For example, response to endogenous stimulus and cell organization and biogenesis were only enriched under a high dose of Zn treatment in Xiangyaxiangzhan (XT2) but not in a low dose of Zn (XT1). These results indicate that a portion of Zn-induced DAS sites is conserved in both fragrant rice cultivars, whereas we do observe genotype-dependent and/or Zn dose-dependent DAS sites.

### Analysis of putative genes involved in regulatory mechanism of 2-AP biosynthesis

**FIGURE 4:**
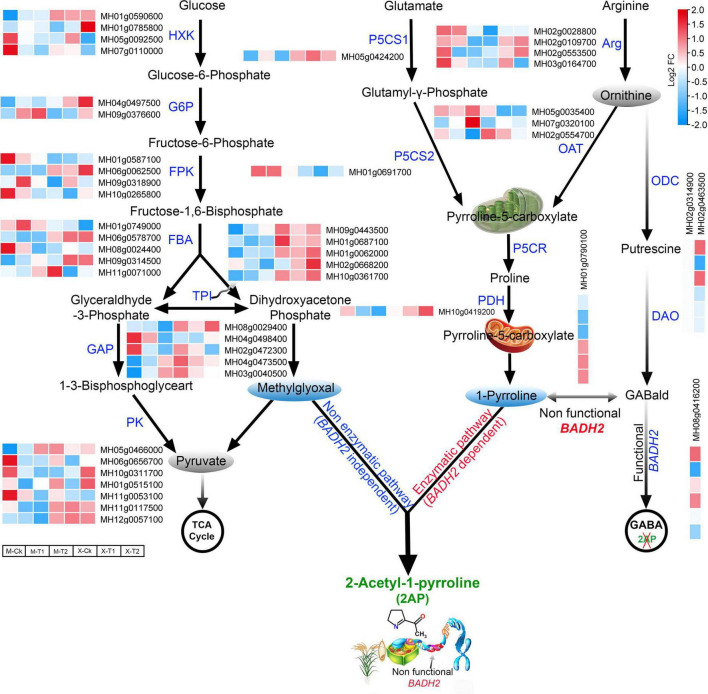
Schematic representation and gene expression summary of 2-AP biosynthesis pathways related genes in Meixiangzhan-2 (M) and Xiangyaxiangzhan (X) cultivars in response to different levels of Zn treatment. Blue, white, and red indicate low, no and high gene expression, respectively.

The amino acids proline, glutamate, and ornithine are the major precursors of 2-AP biosynthesis (Yoshihashi et al., 2002). Therefore, keeping in mind the above key precursors and KEEG pathway associated with 2-AP biosynthesis, we re-analyzed our RNA-seq data of Meixiangzhan-2 and Xiangyaxiangzhan cultivars. A total of 138 differentially expressed genes (DEGs) related to the 2-AP biosynthesis pathway were identified and presented *via* a heatmap (Supplementary Figure 1 and Supplementary Table 4). 19 genes involved in the production of glutamate, 23 genes involved in the production of proline and 11 genes involved in the production of ornithine precursors were identified in both cultivars. In addition to these precursors, we found that foliar Zn spray had significant effects on various rice-specific transcription factors. For example, 17 WRKY, 11 MYB and 8 BHLH transcription factors were upregulated in response to Zn treatment and are also presented in Supplementary Figure 1. Among the 138 DEGs, we further selected 44 key regulatory pathway genes including *MH01g0610600* (Glutamate 5-kinase), *MH03g0702000* (Glutamate dehydrogenase), *MH06g0041100* (Glutamate form-imidoyl transferase), *MH08g0529000* (Glutamate-1-semialdehyde 2,1-aminomutase), *MH02g0604900* (Glutamine synthetase) from glutamate precursors; *MH02g0028800* (Arginine-rich splicing factor RS41) *MH05g0035400* (Acetylornithine aminotransferase), *MH02g0554700* (Acetylornithine deacetylase) from ornithine precursors; and *MH01g0791700* (Proline-rich protein), *MH01g0528900* (Proline-rich receptor-like protein kinase), and *MH10g0419200* (Proline dehydrogenase), *MH05g0424200* (Δ1-pyrroline-5-carboxylate synthase) and *MH01g0790100* (Pyrroline-5-carboxylate reductase) from proline precursors. The results showed that these selected 44 genes were differentially expressed in both cultivars (Figure 4). Furthermore, we surveyed the glycolysis pathway to identify the methylglyoxal biosynthesis genes that were differentially expressed in the regulatory mechanism of 2-AP biosynthesis genes. Among the glycolysis pathways, *MH01g0590600* (Hexokinase-6), *MH04g049750*0 (G6PDH, glucose-6-phosphate 1-dehydrogenase), *MH01g0749000* (Fructose-bisphosphate aldolase), *MH01g0062000* (Triosephosphate isomerase), *MH08g0029400* (Glyceraldehyde-3-phosphate dehydrogenase) and *MH11g0053100* (Pyruvate kinase 1, cytosolic) were mainly included in the upregulated genes. Overall, these findings propose the interconnections between the above pathways regulating 2-AP biosynthesis in fragrant rice. We summarized our findings in Figure 4.

We further predicted protein-protein interactions among 2-AP biosynthesis pathway-related genes to understand their regulatory roles. Most of the proteins revealed strong protein-protein interaction networks, as depicted in Supplementary Figure 2. The K-means algorithm was used for protein clustering into three colors. The results showed that major interaction of the pathway genes (indicated in green color) with the enzymes of glycolysis pathways such as glyceraldehyde-3-phosphate dehydrogenase, phosphoglycerate kinase, triosephosphate isomerase, and betaine aldehyde dehydrogenase. They also showed their interaction with proline biosyntheses, such as Δ1-pyrroline-5-carboxylate synthetase (P5CS), ornithine aminotransferase, and ornithine decarboxylase, proline dehydrogenase, pyrroline-5-carboxylate synthetase and pyrroline-5-carboxylate reductase in Krebs cycle. The results suggest the crosstalk and the coordinated role of these genes in the plant cell during 2-AP biosynthesis under Zn supply.

### Zn induces the SNPs in 2-AP biosynthesis pathway-related genes

Meixiangzhan-2 and Xiangyaxiangzhan showed differential expression of genes involved in the 2-AP biosynthesis pathway (Figure 4). We, therefore, investigated if Meixiangzhan-2 and Xiangyaxiangzhan cultivars differ in SNPs specifically in the genes involved in the 2-AP biosynthesis pathway and if Zn treatment induces the SNPs in both cultivars and that might affect their expression levels (Table 1 and Supplementary Table 5). Indeed, the results showed that there are genotype-specific SNPs in the 2-AP biosynthesis pathway-related genes. For example, *MH06g0082100* has two SNPs and is found specifically in Xiangyaxiangzhan, while *MH01g0062000* has four SNPs and is specifically found in Meixiangzhan-2. Interestingly, we also observed that Zn differently induces the frequency of editing of SNPs in both cultivars. For example, as compared to the control, *MH06g0082100* showed a higher frequency of editing of the two identified SNPs after Zn treatment in Xiangyaxiangzhan, while *MH01g0062000* showed a lower editing frequency of four identified SNPs in Meixiangzhan-2. Furthermore, we also found Zn-induced SNPs in 2-AP biosynthesis pathway-related genes in both cultivars as these SNPs were not present in the control. For example, *MH12g0449300* has two SNPs induced by Zn in both cultivars. Similarly, some other genes related to the 2-AP biosynthesis pathway share common SNPs between Meixiangzhan-2 and Xiangyaxiangzhan but display a different level of editing frequencies. We further investigated whether the presence of SNPs alters the gene expression in response to Zn treatment. The results showed that, among 2-AP-related genes that carry SNPs, the expression of *MH06g0055000* increased in response to a high dose of Zn as compared with control in both cultivars. However, the expression of *MH04g0174500* only increased in Xiangyaxiangzhan as compared to the control (Supplementary Table 6). It is also important to mention that only a few genes related to the 2-AP biosynthesis pathway carry SNPs. Together, these results highlight the presence of genotype-dependent and Zn-induced SNPs in the 2-AP biosynthesis pathway.

**TABLE 1:** Summary of SNPs in the genes related to the 2-AP biosynthesis pathway in Meixiangzhan-2 and Xiangyaxiangzhan cultivars in response to Zn treatment.

Variety	Treatment	Gene	Chromosome	Loci	Editing type	AV frequency	Structure type	Function type
Xiangyaxiangzhan	Zn	MH04g0174500	Chr04	8469174	G- > A	0.55	Exonic	Non-synonymous SNV
	CK	MH06g0082100	Chr06	2924191	T- > C	0.3304	Exonic	Synonymous SNV
	Zn	MH06g0082100	Chr06	2924191	T- > C	0.46105	Exonic	Synonymous SNV
	CK	MH06g0082100	Chr06	2927144	A- > C	0.3421	Exonic	Non-synonymous SNV
	Zn	MH06g0082100	Chr06	2927144	A- > C	0.49405	Exonic	Non-synonymous SNV
Meixiangzhan-2	CK	MH01g0062000	Chr01	2436326	T- > G	0.83395	Exonic	Non-synonymous SNV
	Zn	MH01g0062000	Chr01	2436326	T- > G	0.7494	Exonic	Non-synonymous SNV
	CK	MH01g0062000	Chr01	2436747	T- > C	0.8666	Exonic	Synonymous SNV
	Zn	MH01g0062000	Chr01	2436747	T- > C	0.7654	Exonic	Synonymous SNV
	CK	MH01g0062000	Chr01	2437254	C- > T	0.6369	Exonic	Synonymous SNV
	Zn	MH01g0062000	Chr01	2437254	C- > T	0.48625	Exonic	Synonymous SNV
	CK	MH01g0062000	Chr01	2437272	A- > G	0.54115	Exonic	Synonymous SNV
	Zn	MH01g0062000	Chr01	2437272	A- > G	0.40075	Exonic	Synonymous SNV
	Zn	MH08g0529000	Chr08	24529097	C- > A	0.89275	Exonic	Non-synonymous SNV
	Zn	MH08g0529000	Chr08	24529236	T- > C	0.897	Exonic	Synonymous SNV
	Zn	MH06g0105200	Chr06	4172952	A- > T	0.50075	Exonic	Non-synonymous SNV
	CK	MH06g0105200	Chr06	4172952	A- > T	0.4239	Exonic	Non-synonymous SNV
	Zn	MH06g0105200	Chr06	4172952	A- > T	0.4822	Exonic	Non-synonymous SNV
	CK	MH06g0105200	Chr06	4173004	C- > G	0.4949	Exonic	Non-synonymous SNV
	Zn	MH06g0105200	Chr06	4173004	C- > G	0.54595	Exonic	Non-synonymous SNV
	CK	MH06g0105200	Chr06	4175485	A- > T	0.523	Exonic	Synonymous SNV
	Zn	MH06g0105200	Chr06	4175485	A- > T	0.46905	Exonic	Synonymous SNV
	CK	MH06g0105200	Chr06	4175518	A- > C	0.4286	Exonic	Synonymous SNV
	Zn	MH06g0105200	Chr06	4175518	A- > C	0.41665	Exonic	Synonymous SNV
	Zn	MH08g0523100	Chr08	24129407	G- > A	0.8822	Exonic	Non-synonymous SNV
	Zn	MH08g0523100	Chr08	24129883	G- > A	0.87845	Exonic	Non-synonymous SNV
	Zn	MH08g0523100	Chr08	24130059	C- > T	0.83365	Exonic	Synonymous SNV
	Zn	MH08g0550000	Chr08	25218199	T- > C	0.64585	Exonic	Synonymous SNV
	Zn	MH05g0523700	Chr05	26849016	G- > A	0.25835	Exonic	Non-synonymous SNV
	Zn	MH06g0055000	Chr06	2047443	C- > T	0.79405	Exonic	Synonymous SNV
	Zn	MH06g0055000	Chr06	2047509	G- > A	0.16415	Exonic	Synonymous SNV
Xiangyaxiangzhan and Meixiangzhan-2	Zn	MH12g0449300	Chr12	24231528	T- > A	0.165	Exonic	Synonymous SNV
	Zn	MH12g0449300	Chr12	24231528	T- > A	0.1655	Exonic	Synonymous SNV
	MCK	MH12g0449300	Chr12	24239919	A- > G	0.2738	Exonic	Synonymous SNV
	MZn	MH12g0449300	Chr12	24239919	A- > G	0.2919	Exonic	Synonymous SNV
	XCK	MH12g0449300	Chr12	24239919	A- > G	0.2604	Exonic	Synonymous SNV
	XZn	MH12g0449300	Chr12	24239919	A- > G	0.23275	Exonic	Synonymous SNV
	MCK	MH11g0042500	Chr11	1626340	A- > G	0.16485	Exonic	Synonymous SNV
	MZn	MH11g0042500	Chr11	1626340	A- > G	0.15805	Exonic	Synonymous SNV
	XCK	MH11g0042500	Chr11	1626340	A- > G	0.18255	Exonic	Synonymous SNV
	XZn	MH11g0042500	Chr11	1626340	A- > G	0.1569	Exonic	Synonymous SNV
	MCK	MH11g0042500	Chr11	1626928	T- > C	0.2335	Exonic	Synonymous SNV
	MZn	MH11g0042500	Chr11	1626928	T- > C	0.1898	Exonic	Synonymous SNV
	XCK	MH11g0042500	Chr11	1626928	T- > C	0.12775	Exonic	Synonymous SNV
	XZn	MH11g0042500	Chr11	1626928	T- > C	0.14665	Exonic	Synonymous SNV

### Validation of 2-AP biosynthesis genes

To validate the expression profiles, qRT–PCR was used to quantify the expression of genes involved in the regulatory mechanism of 2-AP biosynthesis (i.e., *TPS9, PDH, GAPC*, and *FBA*) or encoding *MYB*/*WRKY*/*BHLH* TFs that were chosen from the RNA-seq data (Supplementary Figure 3). There was a high correlation between the FPKM values obtained by RNA-seq and the qRT–PCR-derived relative gene expression values. Some of the tested genes showed similar expression patterns in both cultivars in response to Zn treatment. For example, *OsFBA, PME21* and *LAT59* showed an increase in expression in response to the high dose of Zn as compared to the control. However, we found that some of the selected genes were differentially regulated in both cultivars in response to Zn treatment. For example, *ALDH3H1, PDH*, and *TPS9* are downregulated in the Meixiangzhan-2 but upregulated in Xiangyaxiangzhan in response to Zn treatments. *WRKY70, GAMYB* and *BHLH62* showed higher expression in both cultivars in response to Zn treatment. Consistent with RNA-seq, different genes showed differential responses, either up or downregulated, as compared to the control during the molecular mechanism of 2-AP biosynthesis.

### 2-AP biosynthesis pathway-related genes and differential alternative splicing sites display little overlap under Zn treatment in a fragrant rice

**FIGURE 5:**
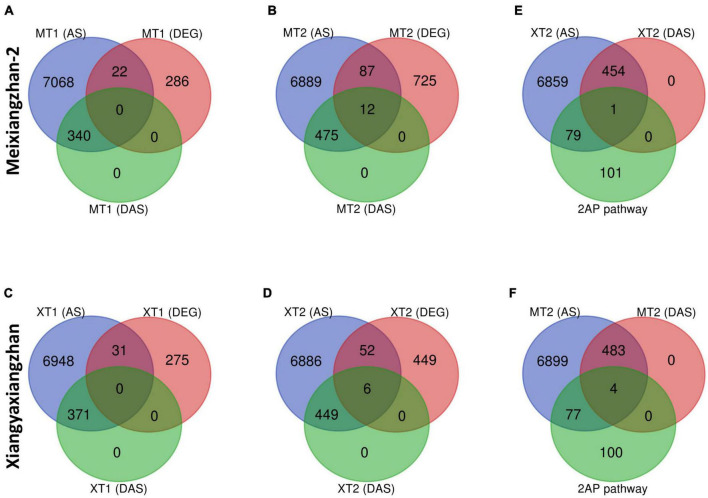
Overlap of differentially alternative spliced (DAS) events with differentially expressed genes (DEGs) in Meixiangzhan-2 (M) and Xiangyaxiangzhan (X) cultivars in response to different levels of Zn treatment. Venn diagram representing the overlap between unique AS sites, DAS events and DEGs in response to different doses of Zn in Meixiangzhan-2 **(A,B)** and Xiangyaxiangzhan **(C,D)** in response to different doses of Zn treatment. **(E,F)** Venn diagram representing the overlap between unique AS sites, DAS events and 2-AP biosynthesis pathway-related genes in Meixiangzhan-2 and Xiangyaxiangzhan.

We next investigated whether there is a correlation between DAS sites and DEGs in both Meixiangzhan-2 and Xiangyaxiangzhan cultivars (Figure 5). Although a small number of DEGs overlapped with total AS sites (Figures 5A–D), only 12 DEGs overlapped with DAS sites in response to a high dose of Zn in Meixiangzhan-2 (MT2), while only 6 DEGs overlapped with DAS sites in response to a high dose of Zn in Xiangyaxiangzhan (XT2). Among the 12 or 6 DEGs that overlapped with DAS sites in Meixiangzhan-2 or Xiangyaxiangzhan (XT2), some are upregulated or downregulated as compared to the control. These observations suggest that Zn-induced DAS sites may contribute to the altered gene expression in fragrant rice. We next investigated if any of the 2-AP pathway-related genes overlapped with DAS sites in response to a high dose of Zn in both cultivars (Figures 5E,F). Although 44% of 2-AP pathway-related genes overlapped with AS sites in response to a high dose of Zn in both Meixiangzhan-2, and Xiangyaxiangzhan cultivars, only 1 and 4 genes overlapped with DAS sites in Meixiangzhan-2, and Xiangyaxiangzhan, respectively. These results indicate that transcriptional regulation and mRNA splicing are controlled by independent mechanisms in fragrant rice.

### Analysis of epigenetic marks at the 2-AP biosynthesis genes

**FIGURE 6:**
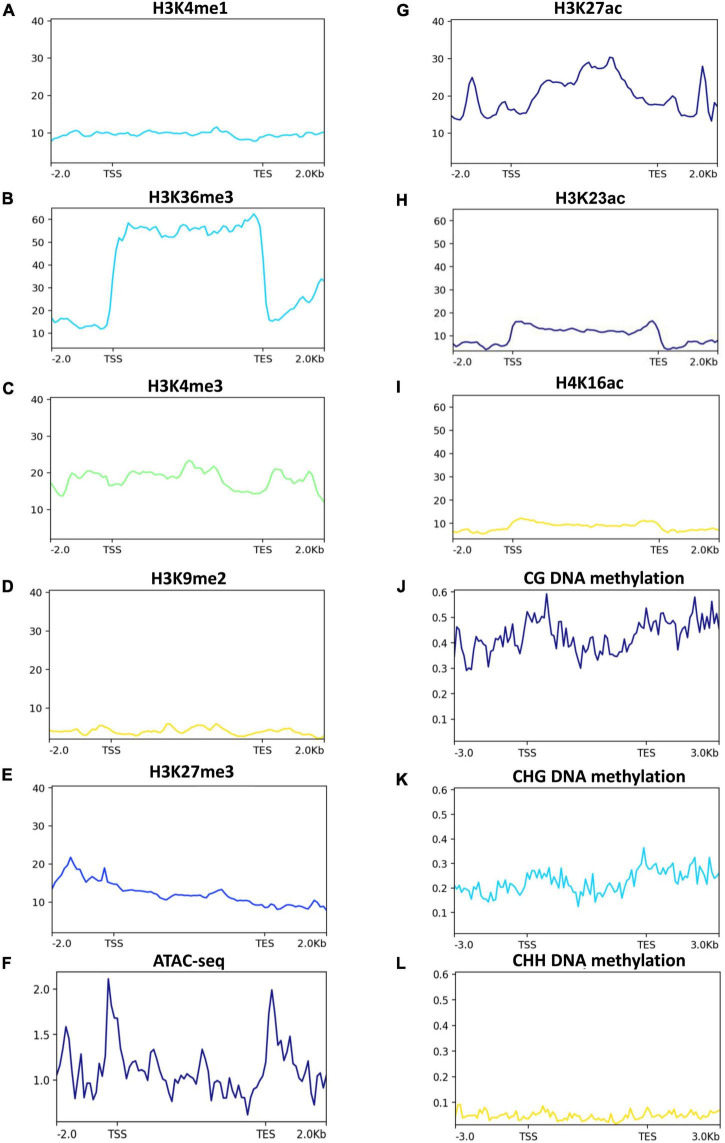
Histone methylation and chromatin accessibility status in 2-AP biosynthesis pathway-related genes in rice. Published data were used to plot histone methylation and ATAC-seq status. H3K4me1, H3K36me, H3K4me3 **(A–C)**, are generally associated with transcriptional activation, while H3K9me2 and H3K27me3 and **(D,E)** are generally associated with transcriptional repression. Chromatin accessibility is determined by Assay for Transposase-Accessible Chromatin using sequencing (I- ATAC-seq) **(F)**. **(G–I)** H3K27ac, H3K23ac and H4K16ac are generally associated with transcriptional activation. While DNA methylation **(J–L)** are generally associated with transcriptional repression.

Epigenetic modifications, such as histone modifications, positioning of nucleosomes and DNA methylation are important components of plants’ gene expression (Berr et al., 2011; Zhang et al., 2018). To investigate if the 2-AP biosynthesis pathway-related genes are under the control of epigenetic modifications, we used publically available rice ChIP-seq of histone modifications and bisulfite-seq of DNA methylation and plotted the data for 2-AP biosynthesis pathway-related genes. Among the histone methylations that are associated with transcriptional activation (H3K4me1, H3K36me3, and H3K4me3), H3K36me3 showed a strong enrichment (Figures 6A–C). Because active histone methylation modifications counteract repressive histone methylation modifications (H3K9me2 and H3K27me3), we observed very low enrichment of H3K27me3 and H3K9me2 at the 2-AP pathway-related genes (Figures 6D,E). These observations suggest that 2-AP biosynthesis pathway-related genes are mainly regulated by active chromatin modifications. Indeed, by using ATAC-seq data, we observed more open chromatin at the transcription start site (TSS) and transcription end site (TES) at 2-AP biosynthesis pathway-related genes (Figure 6F). Consistent with these observations, H3K27ac, H3K23ac and H4K16ac are also enriched over 2-AP biosynthesis pathway-related genes but to a lower level than H3K36me3 (Figures 6G–I). DNA methylation is associated with gene repression (Zhang et al., 2018), and we observed that all three types of DNA methylation (CG, CHG, and CHH) are present at 2-AP biosynthesis pathway-related genes (Figures 6J–L). However, as expected CG DNA methylation levels are higher than CHG and CHH DNA methylation. Together, these results suggest that the 2-AP biosynthesis pathway-related genes are epigenetically regulated.

### Transcriptional regulation of silicon, iron, zinc and other heavy metals transporters genes in response to Zn treatment

**FIGURE 7:**
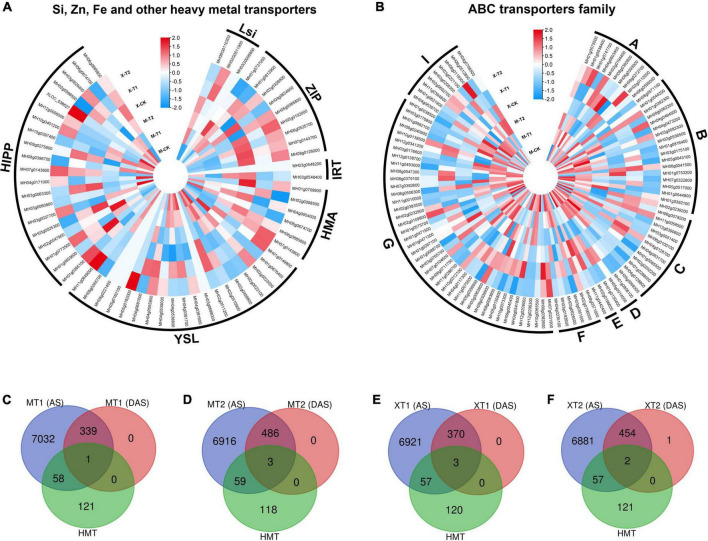
Silicon, Zinc, Iron and other have metals transporters genes identified in DEGs in Meixiangzhan-2 (M) and Xiangyaxiangzhan (X) cultivars in response to different levels of Zn treatment. Blue, white, and red indicate low, no and high gene expression, respectively **(A,B)**. While, Venn diagram representing the overlap between AS sites, DAS events and HMTs in response to different doses of Zn in Meixiangzhan-2 **(A,B)** and Xiangyaxiangzhan **(C–F)**.

Zn has been shown to facilitate the transport of heavy metals, such as lead (Pb) and cadmium (Cd), to aerial parts of the plants (Shafiq et al., 2020), and the specificity of heavy metal transporters has been an outstanding question in the field. Recent advances in OMICs enabled us to improve the toxic metal tolerance (Raza et al., 2022), therefore, we investigated whether the application of Zn could alter the gene expression of heavy metal transporters in fragrant rice. By analyzing RNA-seq data, we identified > 160 heavy metal transporters and heavy metal-related genes, including silicon transporters (*Lsi* 3 genes), iron transporters (*IRT* 2 genes), Zn transporters (9 *ZIP* genes), Heavy metal associated ATPase (*HMA* 6 genes), metal-nicotianamine transporter (*YSL* 19 genes), heavy metal-associated isoprenylated plant protein (*HIPP* 20 genes) and ABC transporters family (ABC family 102 genes) (Figures 7A,B). Indeed, the results showed that both cultivars, Meixiangzhan-2 and Xiangyaxiangzhan showed differential expression of genes including silicon, iron, Zn and other heavy metal transporters genes as compared with their controls (Figure 7). Interestingly, *OsLsi6* (*MH06G0110300*), showed higher expression in Meixiangzhan-2 and lower expression in Xiangyaxiangzhan as compared to the control. However, the expression of *OsLsi1* (*MH02G0511900*) was increased in both cultivars compared to the control in response to Zn treatment. Similarly, the expression level of *ZIP2* (*MH03g0326800*) was higher in both cultivars as compared to the control, while *ZIP8* (*MH07g0143700*) expression was increased in Meixiangzhan-2 and decreased in Xiangyaxiangzhan, respectively. In addition, other heavy metal transporters genes, including *HMA1* (*MH06g0674700*), *YSL8* (*MH02g0023100*), *HIPP6* (*MH03g0060800*) and *ABCC4* (*MH01g0288000*) were downregulated, whereas *YSL7* (*MH02g0023000*), *HIPP26* (*MH01g0773500)*, *ABCG43* (*MH07g0392600*) were upregulated as compared to the control in both cultivars in response to Zn treatment, respectively. On the other side, *HMA2* (*MH06g0685800*), *YSL6* (*MH04g0381600*), *HIPP39* (*MH10g0397400*) and *ABCA2* (*MH08g0392800*) showed their differential expression in both cultivars in response to Zn treatment. Overall, these results indicate the unspecific regulation of heavy metal transporters in response to Zn.

### Post-transcriptional regulation of silicon, iron, zinc and other heavy metals transporters genes in response to Zn treatment

**FIGURE 8:**
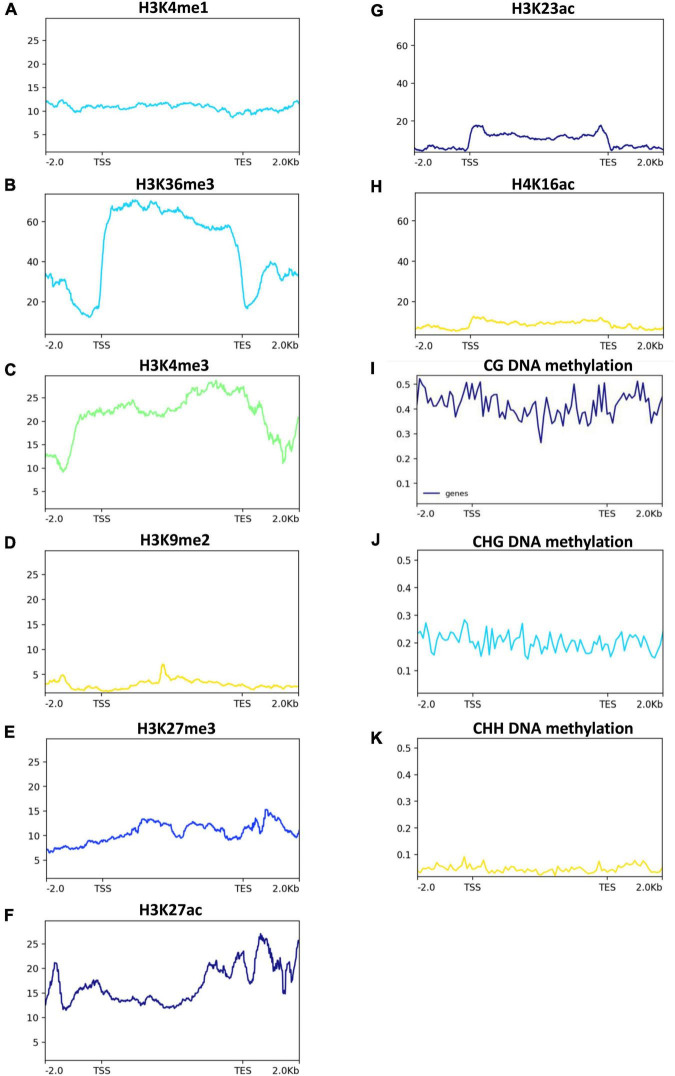
Histone acetylation and DNA methylation status in 2-AP biosynthesis pathway-related genes in rice. Published data were used to plot histone acetylation and DNA methylation status. H3K4me1, H3K36me, and H3K4me3 **(A–C)**, are generally associated with transcriptional activation. While H3K27me3 and H3K9me2 **(D,E)** are generally associated with transcriptional repression. H3K27ac, H3K23ac and H4K16ac **(F–H)**, are generally associated with transcriptional activation. While DNA methylation **(I–K)** are generally associated with transcriptional repression.

We next investigated the post-transcriptional regulation of heavy metal transporters and related genes in response to Zn. Among the > 160 heavy metal transporters and heavy metal-related genes, ∼33% of genes showed the presence of AS events in both cultivars in response to Zn treatment (Figures 7C–F), suggesting the post-transcriptional regulation of heavy metal transporter genes. However, it appears that the remaining ∼67% of genes did not exhibit AS events in response to Zn. Surprisingly, only few genes, mainly from the ABC transporter family, carry significant AS events in response to Zn treatment. *ABCI11* (*MH11g0394800*) from MT1, while *ABCG22* (*MH03g0063800*), *ABCI20* (*MH03g0208300*), *ABCB11* (*MH01g0544200*) from MT2 showed differential alternative splicing in response to Zn in Meixiangzhan-2. Similar to Meixiangzhan-2, *ABCC3* (*MH01g0085500*), *ABCF3* (*MH02g0706000*), *ABCC10* (*MH06g0078200*) from XT1, while *ABCG22* (*MH03g0063800*) and *ABCC3* (*MH01g0085500*) from XT2 showed differential alternative splicing events in Xiangyaxiangzhan. Consistent with 2-AP pathway, heavy metal transporters are enriched with H3K36me3 and H3K4me3 as compared to H3K9me and H3K27me3 (Figures 8A–E). These observations suggest that heavy metal transporters are mainly regulated by active chromatin modifications. Consistent with these observations, H3K27ac, H3K23ac and H4K16ac are also enriched over heavy metal transporters but to a lower level than H3K36me3 (Figures 8F–H). We also observed that all three types of DNA methylation (CG, CHG, and CHH) are present at heavy metal transporters (Figures 8I–K). However, as expected CG DNA methylation levels are higher than CHG and CHH DNA methylation. Together, our analysis revealed the transcriptional and post-transcriptional regulation of heavy metal transporters in fragrant rice in response to Zn.

## Discussion

Plants have evolved complex systems to control their growth and development in response to mineral nutrient availability in the changing environment. Zn is important for the physiological and biochemical functions of plants. Therefore, previous research in plants was focused on transcriptional changes in response to Zn to identify the molecular pathways and the transcription factors that control these pathways (Schvartzman et al., 2018; Roda et al., 2020; Bao et al., 2021). However, post-transcriptional regulation of gene expression in response to Zn is largely ignored. In this study, we identified AS events in two fragrant rice cultivars (Meixiangzhan-2 and Xiangyaxiangzhan) in response to different regimes of Zn. By comparing AS in Meixiangzhan-2 and Xiangyaxiangzhan, we found that some DAS sites are conserved in both cultivars. Consistent with this observation, we observed that genes carrying these shared DAS sites are enriched in physiological processes, metabolism and cellular process in both cultivars (Figure 3). However, we do observe genotype-dependent and Zn-dose dependent AS in both cultivars (Figure 2). In this study, we used the ribo-minus RNA sequencing method, which generally results in a higher RI ratio as compared to other AS types (Cui et al., 2010; Zhao et al., 2014). Indeed, one of the most abundant AS type found in Meixiangzhan-2 and Xiangyaxiangzhan belongs to RI, while MXE was the least abundant in both cultivars. Furthermore, this ratio of AS abundance has already been reported in rice in response to the Cd stress (He et al., 2015; Dong et al., 2018). Interestingly, we observed that DAS sites increase by an increase in the dose of Zn in both cultivars. We also observed more DEGs in Meixiangzhan-2 (824 DEGs) and Xiangyaxiangzhan (517 DEGs) in response to the high dose of Zn as compared to the low dose of Zn (308 DEGs in Meixiangzhan-2 and 316 DEGs in Xiangyaxiangzhan). These results indicate that Zn affects the AS and gene transcription in a dose-dependent manner. However, DAS and DEGs showed a very little overlap in both cultivars and GO analysis of DAS and DEGs (Figure 3 and Supplementary Figure 3) also revealed the enrichment of different pathways in both cultivars. For example, the physiological process is the top enriched pathway in DAS, while plant-pathogen interaction was the top enriched pathway in DEGs of Meixiangzhan-2 (Figure 3 and Supplementary Figure 4). A similar finding was reported in Arabidopsis and rice (Li et al., 2013; Nishida et al., 2017; Dong et al., 2018). This suggests that mRNA splicing and gene expression are regulated by independent mechanisms, which are mediated by splicing factors and transcription factors, respectively (Staiger and Brown, 2013). However, further studies are required to investigate the post-transcriptional regulation of Zn-induced DEGs and AS events in fragrant rice.

Fragrant rice (*Oryza sativa* L.) is eminent around the world due to its unique aroma and 2-AP is responsible for the aroma of fragrant rice (Poonlaphdecha et al., 2016). Several factors affect 2-AP biosynthesis in fragrant rice. However, the method by which fragrance is created and transported to grain is complex, including multiple genes and transcription factors. Numerous reports have demonstrated that micronutrients and fertilizers can increase 2-AP production in fragrant rice (Li et al., 2016; Mo et al., 2016, 2017; Lei et al., 2017; Luo et al., 2019b). It was reported that exogenous application of Zn at heading stages enhances 2-AP biosynthesis in various genotypes of rice (Luo et al., 2019a). Previously, it was revealed that the 2-AP biosynthesis pathway involved precursors and intermediates (proline, P5C, 1-pyrroline, methylglyoxal, and GABA) as well as enzymes (PDH, P5CS, OAT, DAO, and BADH2) (Huang et al., 2008; Poonlaphdecha et al., 2012; Ghosh and Roychoudhury, 2018). Therefore, we compared the RNA-seq data of Meixiangzhan-2 and Xiangyaxiangzhan to understand the molecular mechanisms behind the Zn-induced 2-AP biosynthesis in fragrant rice’s flag leaves. Among the DEGs, important aroma-related enzyme genes, such as *TPS, GAPC, LAT59, FBA, P5CS, P5CR*, and *PDH*, were significantly upregulated in response to Zn. However, we observed that the expression of a few 2-AP biosynthesis-related genes is differentially regulated in both cultivars in response to Zn. Interestingly, we found that the editing frequency of SNPs present in the 2-AP biosynthesis pathway-related genes is also sometimes different between two cultivars in response to Zn. However, further studies are required to understand whether these Zn-induced SNPs have an impact on gene expression.

Furthermore, we found very little overlap among DAS sites and 2-AP pathway genes (Figures 5E,F), which suggests that the 2-AP pathway is regulated independently of splicing factors. Consistent with this, we found that foliar Zn spray had significant effects on various rice-specific transcription factors in our investigation, such as *MH11g0026200* (*WRKY*), *MH11g0318400* (*bHLH*), and *MH06g0666100* (*MYB*) were significantly upregulated (Supplementary Figure 1). A previous study also reported that the *MYB/bHLH* transcription factor also regulates floral scent metabolism by binding to enzyme promoter regions and supports our results (terpene synthases) (Deluc et al., 2006; Reddy et al., 2017; Abbas et al., 2021). Furthermore, rice plants overexpressing *OsMYB48-1* had greater levels of *OsP5CS1* and *OsP5CS2* expression and accumulated more proline during drought conditions (Xiong et al., 2014). Our data highlight a positive relationship between transcription factors and 2-AP biosynthesis under Zn treatment, and we speculate that the 2-AP pathway is transcriptionally regulated instead of post-transcriptionally regulated by AS. However, it is possible that 2-AP biosynthesis pathway is regulated by epigenetic modifications in response to Zn. Although the regulation of epigenetic modifications in response to Zn is at infancy, there are few reports that highlight Zn may induce epigenetic modifications in plants. Zn treatment regulates the expression of histone acetyltransferases, histone deacetylates, and DNA methyltransferases in plants, suggesting that Zn may control the gene expression through epigenetic modifications (Imran et al., 2019, 2020; Shafiq et al., 2019, 2020). Indeed, Zn has been reported to alter DNA methylation at the promoter of Zn transporters, which led to the change in its gene expression (Shafiq et al., 2019, 2020). Similarly, a connection between histone acetylation and Zn transporter regulation in response to Zn has been suggested (Shafiq et al., 2020). Our results showed the presence of multiple epigenetic modifications on the 2-AP biosynthesis-related genes in the rice genome (Figure 6). H3K36me3 seems to be the most abundant modification on the 2-AP biosynthesis-related genes. However, further studies are required to elucidate whether Zn regulates the epigenetic landscape of the 2-AP biosynthesis-related genes.

Heavy metal stress affects the fitness, survival and yield of crop plants during the course of their development by impairing the molecular, biochemical and physiological processes (Babst-Kostecka et al., 2016). The heavy metal transporters lack specificity and generally respond to more than one metal. Consistent with this, we found that foliar Zn spray had significant effects on various rice-specific metal transporters in our investigation such as *ZIP2 (MH03g0326800)*, *YSL7 (MH02g0023000)*, and *ABCG43 (MH07g0392600)* that were significantly upregulated in both cultivars (Figures 7A,B). It has been reported that silicon mitigate abiotic stress (Mir et al., 2022) and assists Zn tolerance in rice by reducing the uptake and translocation of excess Zn (Gu et al., 2012). Consistently, *Lsi1 (MH06G0110300)* and *Lsi6 (MH02G0511900*), transporters of silicon, were differentially regulated in both cultivars in response to Zn treatment, suggesting a role of silicon in Zn tolerance in fragrant rice. We found that ABC- transporters were mainly regulated in response to Zn in both cultivars (Figure 7B). ABC proteins have multiple functions in plants with a range of substrates, including toxic chemicals, hormones, pigments, secondary metabolites for defense, reactive oxygen species-related compounds and lipidic molecules (Do et al., 2021). In Arabidopsis, the tonoplast-localized ABCCs, such as *AtABCC1*, *AtABCC2*, and *AtABCC3*, take part in transporting phytochelatins and their complexes with As, Cd, Mn, and Zn inside the vacuole to provide metal tolerance (Song et al., 2010; Brunetti et al., 2015). By analyzing the AS events on these metal transporters, we found that around 33% of metal transporters have AS events in both cultivars, suggesting the post-transcriptional regulation of these heavy metal transporters. However, only a few ABC-transporters were differentially spliced in response to Zn treatment. For example, *ABCC3* (*MH01g0085500*) has been differentially spliced in response to the Zn regime in the Xiangyaxiangzhan. Similarly, *ABCG22* (*MH03g0063800*), normally involved in abscisic acid signaling, was differentially spliced in Meixiangzhan-2. Our results showed the presence of multiple epigenetic modifications on the heavy metal transporters in the rice genome and H3K36me3 seems to be the most abundant modification (Figures 8). However, further studies are required to investigate the transcriptional and post-transcriptional regulation of heavy metal transporters in fragrant rice.

## Conclusion

Overall, this study provides evidence of the AS in fragrant rice in response to Zn treatment and highlights that the genes involved in 2-AP biosynthesis pathway and heavy metal transport may also be post-transcriptionally regulated through AS and epigenetic modifications.

## Data availability statement

The datasets presented in this study can be found in the online repository: https://www.ncbi.nlm.nih.gov/sra/PRJNA673016.

## Author contributions

MI, SS, and XT conceived and designed the experiment. MI, SS, SI, GB, AG, ED, EW, SP, and ZM performed the experiments and did the formal analysis. MI and SS wrote the original draft. SS and XT revised and finalized the manuscript. All authors endorsed the final version of the manuscript.

## Footnotes

1
https://rice-genome-hub.southgreen.fr

2
http://rice.hzau.edu.cn/

3
http://bis.zju.edu.cn/

## Supplementary material

Supplementary Figure 1 2-AP biosynthesis genes and the TFs identified in DEGs in Meixiangzhan-2 (M) and Xiangyaxiangzhan (X) cultivars in response to different levels of Zn treatment. Blue, white, and red indicate low, no and high gene expression, respectively.

Supplementary Figure 2 Network analysis of 2-AP biosynthesis pathways-related proteins.

Supplementary Figure 3 Validation of key genes involved in 2-AP biosynthesis through qPCR in Meixiangzhan-2 (M) and Xiangyaxiangzhan (X) cultivars in response to different levels of Zn treatment. *Actin* was used as an internal control.

Supplementary Figure 4 KEGG analysis of differentially expressed Genes (DEGs) in Meixiangzhan-2 (M) and Xiangyaxiangzhan (X) cultivars in response to different levels of Zn treatment.

Supplementary Table 1 List of primers used in the study.

Supplementary Table 2 List of AS events in both cultivars in response to different levels of Zn.

Supplementary Table 3 The comparison of alternative splicing (AS) events in Meixiangzhan-2 (MT1 vs MT2) and Xiangyaxiangzhan (XT1 Vs XT2) in response to Zn treatment.

Supplementary Table 4 List of DEGs in both cultivars in response to different levels of Zn.

Supplementary Table 5 List of SNPs identified in both cultivars in response to different levels of Zn.

Supplementary Table 6 Expression pattern of genes that possess SNPs in response to Zn treatment.
