# Supplementary figures and images for: Post-transcriptional regulation of 2-acetyl-1-pyrroline (2-AP) biosynthesis pathway, silicon, and heavy metal transporters in response to Zn in fragrant rice
Source: Front Plant Sci. 2022 Aug 17;13:948884. doi: 10.3389/fpls.2022.948884 (PMC9428631; doi:10.3389/fpls.2022.948884)

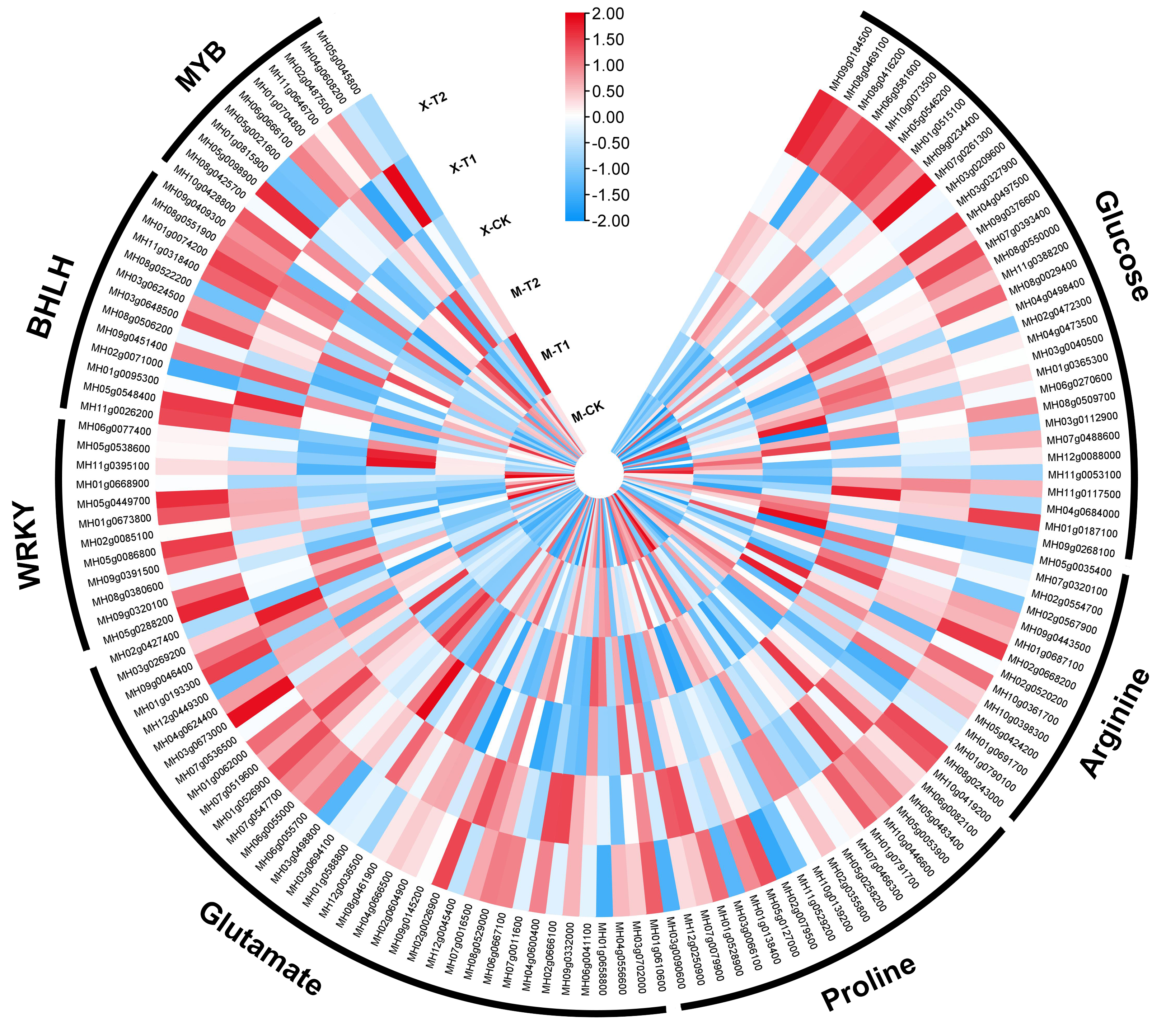

Supplement: Supplementary Figure 1 — 2-AP biosynthesis genes and the TFs identified in DEGs in Meixiangzhan-2 (M) and Xiangyaxiangzhan (X) cultivars in response to different levels of Zn treatment. Blue, white, and red indicate low, no and high gene expression, respectively. [file Image_1.JPEG]

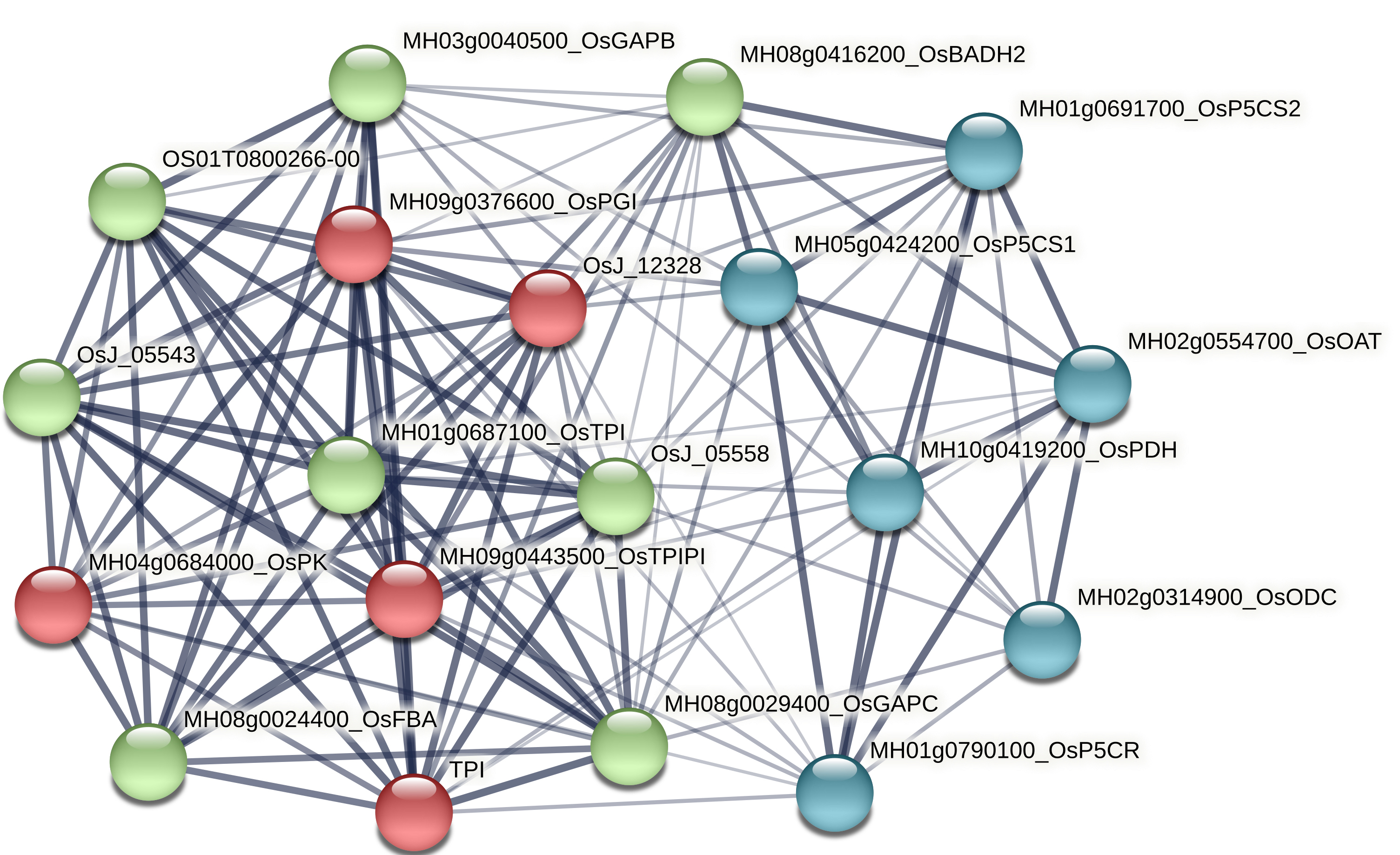

Supplement: Supplementary Figure 2 — Network analysis of 2-AP biosynthesis pathways-related proteins. [file Image_2.JPEG]

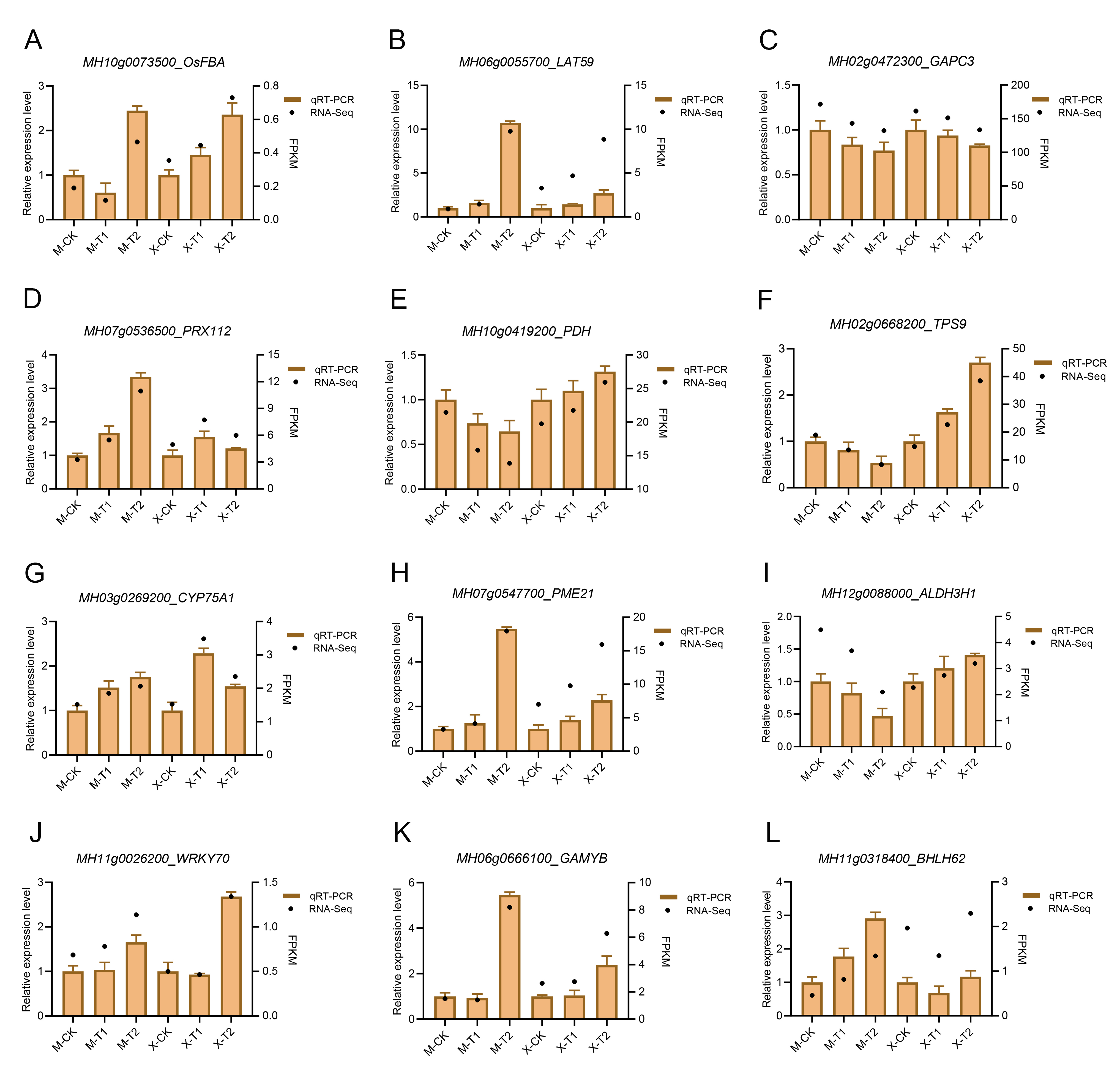

Supplement: Supplementary Figure 3 — Validation of key genes involved in 2-AP biosynthesis through qPCR in Meixiangzhan-2 (M) and Xiangyaxiangzhan (X) cultivars in response to different levels of Zn treatment. Actin was used as an internal control. [file Image_3.JPEG]

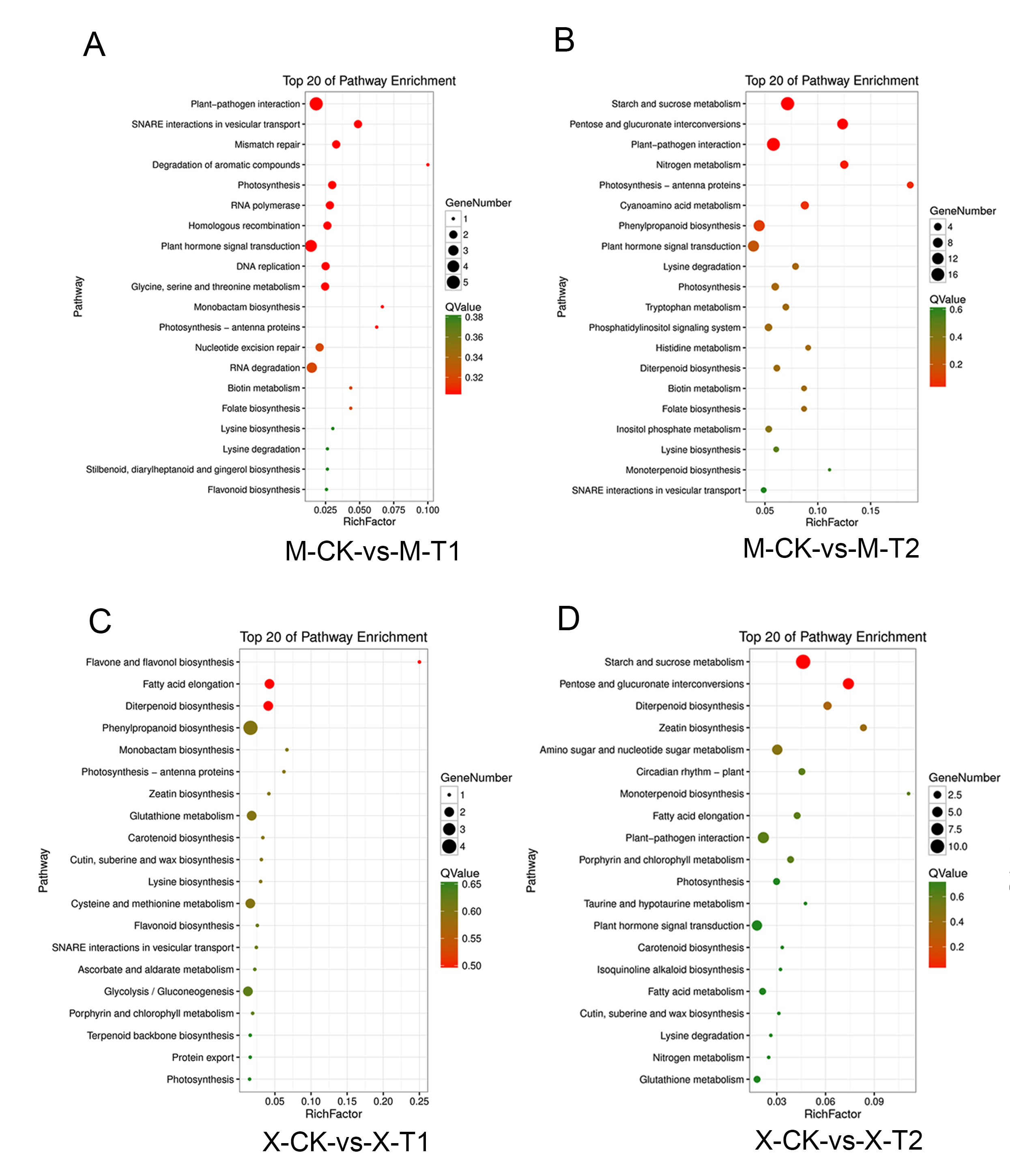

Supplement: Supplementary Figure 4 — KEGG analysis of differentially expressed Genes (DEGs) in Meixiangzhan-2 (M) and Xiangyaxiangzhan (X) cultivars in response to different levels of Zn treatment. [file Image_4.JPEG]
